# Supplementary material for: Global Potential Geographical Distribution of the Southern Armyworm (Spodoptera eridania) under Climate Change
Source: Biology (Basel). 2023 Jul 23;12(7):1040. doi: 10.3390/biology12071040 (PMC10376329; doi:10.3390/biology12071040)
Supplement: Supplementary file 1 [file biology-12-01040-s001.zip › biology-2450827-supplementary.pdf]

Supplementary

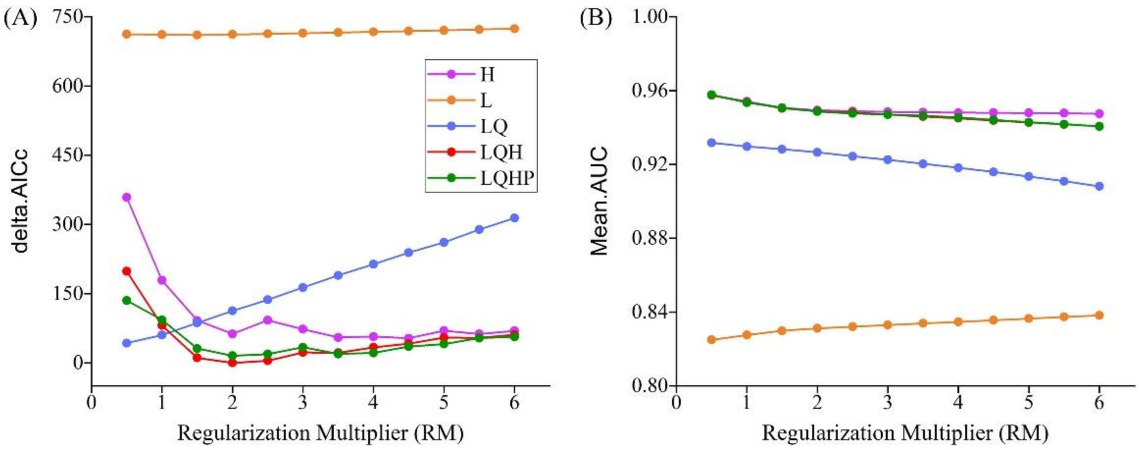

Figure S1. Model optimization results under different parameters

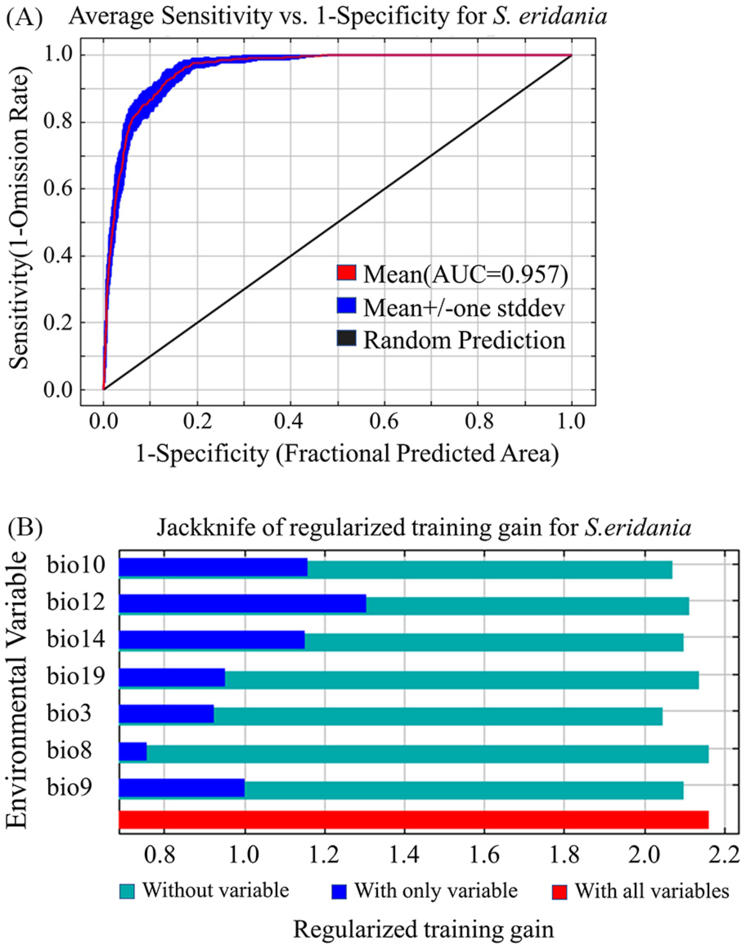

Figure S2. MaxEnt model performance and variable contributions for a predictive model of the global distribution of *Spodoptera eridania*. (A) Receiver operating characteristic (ROC) curve averaged over the replicate runs; (B) Jackknife test of variable importance averaged over the replicate runs.

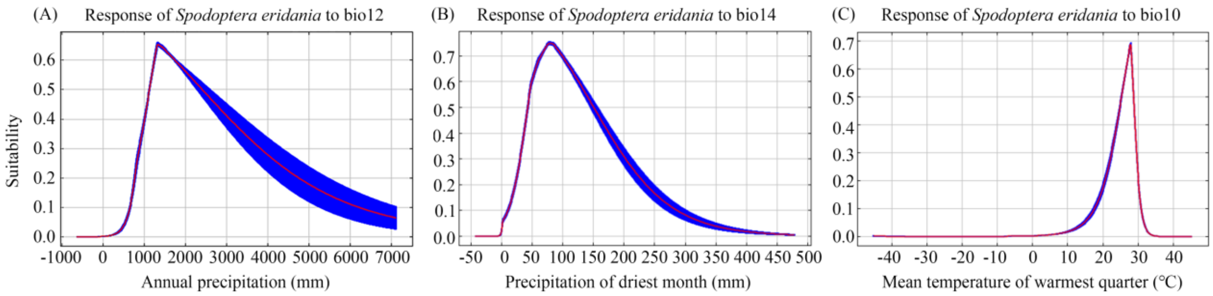

Figure S3. Response curve of the important bioclimatic variables.

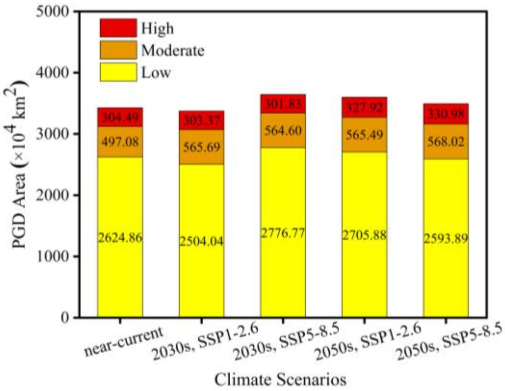

Figure S4. Area of potential geographical distribution of different fitness classes.

Table S1 Spatial variation in potential geographical distribution area (×10^4km^2) for *Spodoptera eridania* under future climate scenarios

Spatial Variation	2030s, SSP1-2.6	2030s, SSP5-8.5	2050s, SSP1-2.6	2050s, SSP5-8.5
Expansion	299.34	396.06	450.93	464.40
Contraction	352.66	179.29	278.07	397.94
Unchange	3073.76	3247.14	3148.36	3028.49

Table S2 Longitude and latitude of the centroid of potential geographical distribution for *Spodoptera eridania*

Continent	near-current	2030s, SSP1-2.6	2030s, SSP5-8.5	2050s, SSP1-2.6	2050s, SSP5-8.5
Africa	1°17′30″N, 20°35′10″E	0°32′42″S, 22°2′58″E	0°7′0″N, 21°13′37″E	0°25′56″S, 22°17′34″E	0°19′51″S, 22°22′15″E
Asia	18°6′7″N, 99°55′53″E	18°50′23″N, 101°25′16″E	19°9′35″N, 100°2′19″E	19°22′36″N, 102°11′45″E	20°12′13″N, 103°29′8″E
Europe	45°9′17″N, 2°13′40″E	45°20′32″N, 3°57′48″E	45°46′42″N, 4°55′51″E	45°50′36″N, 4°41′48″E	45°52′49″N, 5°17′34″E
North America	31°5′45″N, 91°24′34″W	32°16′43″N, 91°10′49″W	32°18′54″N, 92°27′43″W	33°2′50″N, 92°14′28″W	33°27′29″N, 91°52′5″W
Oceania	27°21′13″S, 147°47′15″E	27°58′53″S, 148°18′52″E	27°25′32″S, 148°9′28″E	27°27′22″S, 148°11′9″E	28°10′25″S, 148°34′38″E
South America	12°32′55″S, 59°14′11″W	12°52′49″S, 59°11′28″W	13°6′38″S, 58°59′14″W	12°59′56″S, 58°59′33″W	12°55′45″S, 59°7′43″W
